# Endothelial CPT2 loss impairs fatty acid oxidation and promotes renal fibrosis

**DOI:** 10.1093/lifemeta/loag017

**Published:** 2026-06-13

**Authors:** Xudong Zhao, Zu-Xi Yu, Brendan M Browne, Wentao Li, Yue A Qi, Jianhua Xiong

**Affiliations:** Department of Urology, Emory University School of Medicine, Atlanta, GA 30322, United States; National Heart, Lung and Blood Institute, National Institutes of Health, Bethesda, MD 20892, United States; Department of Urology, Emory University School of Medicine, Atlanta, GA 30322, United States; Winship Cancer Institute of Emory University, Atlanta, GA 30322, United States; Department of Environmental Health Science, College of Public Health, University of Georgia, Athens, GA 30602, United States; Center for Alzheimer’s and Related Dementias, National Institute on Aging and National Institute of Neurological Disorders and Stroke, National Institutes of Health, Bethesda, MD 20892, United States; Department of Urology, Emory University School of Medicine, Atlanta, GA 30322, United States; Winship Cancer Institute of Emory University, Atlanta, GA 30322, United States

Dear Editor,

Mitochondrial fatty acid β-oxidation (FAO) is a major pathway for long-chain fatty acid catabolism and systemic energy homeostasis [1]. During fasting or other metabolic stresses, fatty acids serve as an important fuel source, and FAO remains a principal energy source for organs with high metabolic demand, including the heart, skeletal muscle, and kidney. In the kidney, FAO defects are clinically associated with kidney fibrosis and rhabdomyolysis-induced acute kidney injury [1, 2], and defective FAO in renal tubular epithelial cells has been shown to drive fibrosis [2]. However, whether endothelial FAO impairment contributes to renal pathology remains unclear.

Since Knoop’s early formulation of β-oxidation as the stepwise two-carbon removal from fatty acids [1], subsequent biochemical and genetic studies have established the enzymes, intermediates, inherited disorders, and disease-causing mutations that define the FAO pathway [3, 4]. Among these components, the carnitine shuttle, mediated by carnitine palmitoyltransferase 1 (CPT1) on the outer mitochondrial membrane and carnitine palmitoyltransferase 2 (CPT2) on the inner membrane, is essential for long-chain fatty acid utilization [4]. Sequential CPT1 and CPT2 activity imports long-chain fatty acids from the cytosol into the mitochondria and enables subsequent β-oxidation (Fig. 1a). Both enzymes are essential components of this pathway, with CPT1 commonly regarded as a key regulatory step in long-chain FAO. Consistent with the importance of the carnitine shuttle, homozygous whole-body deletion of the liver isoform CPT1a is lethal in mice [5].

**Figure 1:**
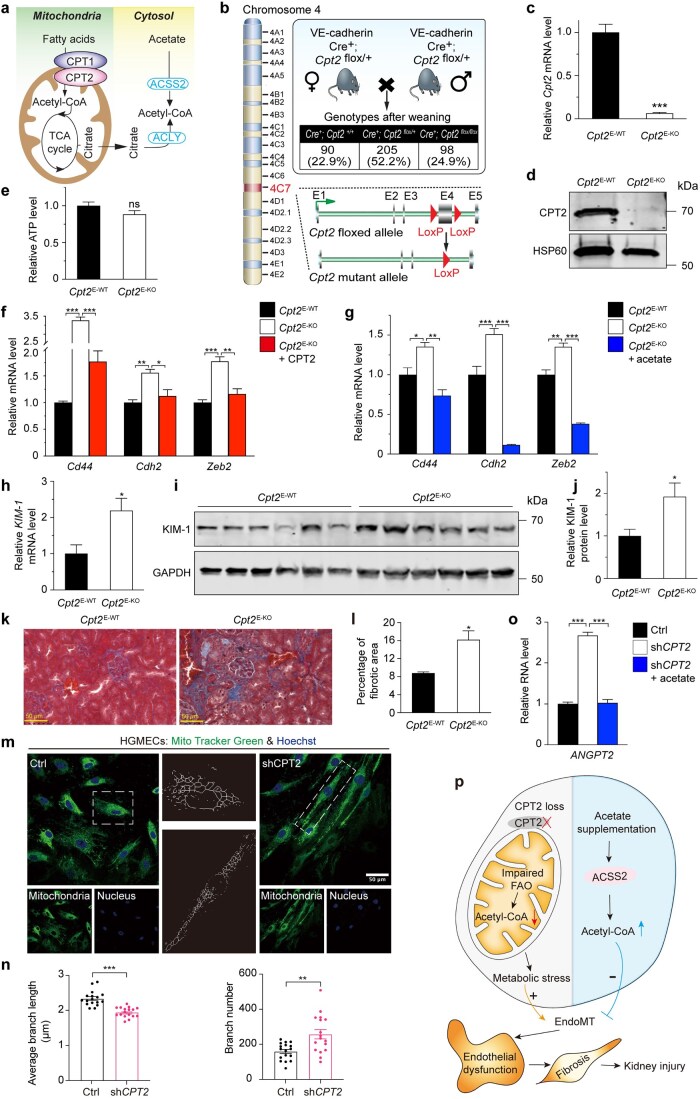
Endothelial FAO regulates acetyl-CoA, EndoMT, and renal injury. (a) Schematic diagram illustrating how FAO generates acetyl-CoA via CPT2 and how acetate replenishes acetyl-CoA through ACSS2. ACLY, ATP citrate lyase; TCA cycle, tricarboxylic acid cycle. (b) Gene-targeting strategy for conditional deletion of *Cpt2* (exon 4 flanked by loxP sites). Viable VE-cadherin Cre-positive offspring from VE-cadherin Cre; *Cpt2*^flox/+^ intercrosses showed expected Mendelian ratios. (c and d) CPT2 expression in primary lung endothelial cells from *Cpt2*^E-WT^ and *Cpt2*^E-KO^ mice, analyzed by qPCR (c) and western blot (d). *n *= 3 independent experiments. (e) Relative ATP levels in *Cpt2*^E-WT^ and *Cpt2*^E-KO^ endothelial cells (*n *= 3). (f) qPCR analysis showing that heterologous *Cpt2* expression suppresses EndoMT marker induction in *Cpt2*^E-KO^ endothelial cells (*n *= 3). (g) qPCR analysis showing that acetate supplementation inhibits EndoMT marker expression in *Cpt2*^E-KO^ cells (*n *= 3). (h–j) Renal injury in *Cpt2*^E-WT^ and *Cpt2*^E-KO^ kidneys, assessed by *KIM-1* mRNA (h; *n *= 5 mice per genotype) and protein levels (i and j; *n *= 6 mice per genotype). (k and l) Representative Masson’s trichrome-stained kidney sections (k) and quantification of fibrotic area (l) from 13–16-month-old male mice (*n *= 3 per genotype). (m) Representative confocal images of HGMECs stained with MitoTracker Green (mitochondria) and Hoechst (nuclei). Middle panels show skeletonized mitochondrial networks used for MiNA analysis. Scale bar, 50 μm. (n) Quantification of mitochondrial network parameters by MiNA analysis, including average branch length and total branch number, calculated from individual cells (*n *= 17). Each dot represents one cell. (o) Relative *ANGPT2* mRNA level measured by qPCR in control (Ctrl), sh*CPT2*, and sh*CPT2 *+ acetate conditions. (p) Schematic model illustrating how loss of FAO promotes EndoMT and contributes to kidney injury. Data are presented as mean ± SEM. Statistical significance was determined by unpaired two-tailed Student’s *t*-test for two-group comparisons and one-way ANOVA with Tukey’s multiple comparison test for comparisons among three or more groups. *P < 0.05; **P < 0.01; ***P < 0.001.

To define the specific contribution of FAO to renal endothelial cell biology, we generated an endothelial-specific *Cpt2* knockout model (*Cpt2*^E-KO^) by crossing floxed *Cpt2* mice with VE-cadherin–Cre transgenic mice [6]. Genotype distribution at weaning followed the expected Mendelian ratios, indicating that endothelial loss of CPT2 did not impair viability (Fig. 1b). As expected, endothelial cells isolated from *Cpt2*^E-KO^ mice exhibited markedly reduced CPT2 expression at both the mRNA and protein levels (Fig. 1c and d). Wild-type (WT) endothelial cells responded to long-chain fatty acid stimulation (palmitate) with a robust increase in oxygen consumption, whereas *Cpt2*-deficient endothelial cells lacked this FAO-dependent respiratory response (Supplementary Fig. S1a). Despite their inability to oxidize long-chain fatty acids, *Cpt2*^E-KO^ endothelial cells maintained normal ATP levels (Fig. 1e), consistent with prior observations that quiescent endothelial cells rely predominantly on glycolysis for basal energetic demands.

Although FAO is classically viewed as an energy-generating pathway, our previous studies indicate that FAO also regulates endothelial cell fate and function [6]. Specifically, FAO inhibition can promote endothelial-to-mesenchymal transition (EndoMT), a process in which endothelial cells lose cobblestone morpho­logy and endothelial markers while acquiring mesenchymal and stem-like features, including upregulation of cluster of differentiation 44 (CD44), cadherin-2 (CDH2), and zinc finger E-box-binding homeobox 2 (ZEB2) [7]. Consistent with this concept, endothelial cells from *Cpt2*^E-KO^ mice showed elevated expression of mesenchymal-associated markers, including *Cd44*, *Cdh2*, and *Zeb2*, compared with WT counterparts (Fig. 1f), suggesting activation of an EndoMT-like transcriptional program. Restoring *Cpt2* expression under a heterologous promoter suppressed EndoMT marker induction in *Cpt2*^E-KO^ endothelial cells (Fig. 1f), supporting the FAO dependence of this phenotype.

Mechanistically, FAO-mediated EndoMT appears to involve acetyl-CoA availability [6] (Fig. 1a). Acetyl-CoA is generated via the tricarboxylic acid (TCA) cycle and serves as the sole donor for protein acetylation, regulating gene expression and protein function [4, 6]. Consistent with this, *Cpt2*^E-KO^ endothelial cells exhibited reduced cellular acetyl-CoA levels [6] (Supplementary Fig. S1b). Supplementation with acetate, which replenishes the acetyl-CoA pool via acyl-CoA synthetase short-chain family member 2 (ACSS2) activity [6, 8, 9], partially suppressed mesenchymal marker expression (Fig. 1g; Supplementary Fig. S1b) in *Cpt2*^E-KO^ endothelial cells, supporting a role for acetyl-CoA in modulating EndoMT in the context of FAO impairment. Together, these observations provide a plausible mechanistic basis whereby the central metabolite acetyl-CoA, acting as a metabolic signaling molecule, links cellular metabolism to epigenetic, transcriptional, and protein level regulation by serving as the sole donor for protein acetylation [3, 5]. This includes histone acetylation, which modulates chromatin accessibility, as well as nonhistone protein acetylation that influences protein stability and activity. In addition, acetylation-dependent regulation of EndoMT-related transcriptional programs, including transforming growth factor-β (TGF-β)/small mothers against decapentaplegic (Smad) signaling (potentially through modulation of Smad7 acetylation), may further contribute to endothelial fate changes, although the relevant downstream substrates remain to be defined [3, 7]. Together, these observations suggest that impaired FAO may disrupt acetyl-CoA-dependent regulatory pathways that promote mesenchymal transition, positioning FAO-derived acetyl-CoA as a candidate upstream metabolic regulator of EndoMT.

To characterize the EndoMT phenotype *in vivo*, we performed lineage tracing using double-fluorescent R26-mTmG reporter mice crossed with *Cpt2*^E-KO^ animals. Single-cell suspensions of kidney cells were analyzed by fluorescence-activated cell sorting (FACS), gating on GFP^+^, CD31^+^, CD45^–^, tdTomato^–^ endothelial cells (Supplementary Fig. S1c). Approximately 3% of endothelial-lineage cells co-expressed the mesenchymal marker α-SMA, and the proportion of CD31^+^/α-SMA^+^ cells was approximately 30% higher in *Cpt2*^E-KO^ mice than in WT controls (Supplementary Fig. S1d and e). These lineage-tracing data suggest that a subset of endothelial-derived cells may acquire mesenchymal-like features *in vivo*, consistent with a partial EndoMT-like process rather than complete lineage conversion. Consistent with these cellular alterations, markers of renal injury were elevated in *Cpt2*^E-KO^ mice, including mRNA and protein levels of kidney injury molecule-1 (KIM-1) (Fig. 1h–j), a well-established indicator of acute and chronic kidney injury [2]. In parallel, histological analysis of Masson’s trichrome-stained kidney sections revealed increased collagen deposition, consistent with a higher degree of renal fibrosis in *Cpt2*^E-KO^ mice (Fig. 1k and l). However, the precise cellular origin of the fibrotic cells cannot be determined from the current experiments; accordingly, these observations highlight an association between endothelial FAO loss and fibrotic remodeling rather than excluding contributions from other renal cell populations. Functionally, urine albumin-to-creatinine ratios were modestly elevated (< 30 µg/mg) in 10–14-month-old *Cpt2*^E-KO^ mice compared with littermate controls (Supplementary Fig. S1f), consistent with early albuminuria and progressive chronic kidney injury. Although age-matched littermate controls were used, age-associated changes in renal physiology may still influence disease susceptibility; accordingly, we cannot exclude the possibility that aging interacts with endothelial CPT2 deficiency to exacerbate fibrotic remodeling. These functional alterations are consistent with EndoMT-associated disruption of endothelial barrier integrity.

Notably, this effect was more pronounced in male mice, whereas female mice showed no significant change, highlighting potential sex-specific susceptibility (Supplementary Fig. S1f). One possible explanation is that sex hormones modulate endothelial meta­bolism and mitochondrial FAO, as hormonal regulation has been shown to shape cellular metabolic programs and susceptibility to tissue injury [5, 10]. Similar sex disparities are observed in renal cell carcinoma (RCC), where incidence and severity are higher in men than in women [10], although the biological basis remains incompletely understood. Our findings do not directly address RCC, but they raise the possibility that sex-dependent endothelial vulnerability may contribute to broader renal pathologies, as loss of endothelial FAO in the *Cpt2*^E-KO^ model promoted EndoMT, reduced acetyl-CoA availability, and triggered molecular and histological evidence of kidney injury. The observation that albuminuria was more prominent in male mice further suggests that sex-specific differences in endothelial metabolism, FAO capacity, or acetyl-CoA-dependent signaling may influence susceptibility to chronic kidney injury and fibroinflammatory microenvironments.

To determine whether the metabolic alterations observed in the mouse model are conserved in human cells, we next examined the role of FAO in human glomerular microvascular endothelial cells (HGMECs). Impaired FAO is a clinically relevant feature of chronic kidney disease (CKD), as kidneys from patients with CKD exhibit reduced expression of FAO-related genes accompanied by lipid accumulation, inflammation, and progressive tubulointerstitial fibrosis [2]. Consistent with this, loss-of-function studies in HGMECs showed that *CPT2* knockdown disrupted mitochondrial network organization, as evidenced by reduced branch length and increased mitochondrial fragmentation in the Mitochondrial Network Analysis (MiNA) of MitoTracker staining (Fig. 1m and n). In parallel, immunofluorescence analysis revealed reduced CD31 staining intensity and compromised endothelial cell–cell junction integrity (Supplementary Fig. S1g and h), reflecting impaired endothelial structural organization and disrupted intercellular interactions critical for vascular barrier function and endothelial–epithelial crosstalk [6]. These mitochondrial and structural changes were accompanied by marked upregulation of endothelial activation and inflammatory genes, including interleukin-6 (*IL6*), C-C motif ligand 2 (*CCL2*), vascular cell adhesion molecule-1 (*VCAM1*,) and intercellular cell adhesion molecule-1 (*ICAM1*), while acetate supplementation partially attenuated these transcriptional responses (Supplementary Fig. S1i), supporting a role for acetyl-CoA availability in regulating endothelial inflammation downstream of FAO impairment. Notably, *CPT2* knockdown also increased angiopoietin-2 (*ANGPT2*) expression (Fig. 1o), a key regulator of endothelial destabilization that antagonizes tyrosine kinase with immunoglobulin-like and EGF-like domains 2 (Tie2) signaling, further indicating compromised vascular integrity. Collectively, these complementary molecular and imaging-based analyses provide convergent evidence that CPT2 deficiency induces mitochondrial structural remodeling, disrupts endothelial junctional organization, and promotes endothelial activation. Based on these findings, we propose a model in which loss of FAO may promote EndoMT and contribute to fibrotic kidney injury (Fig. 1p).

Kidney fibrosis represents a final common pathway of chronic renal injury, and EndoMT has emerged as a key contributor to fibrotic remodeling [11]. Our results support a model in which impaired FAO diminishes acetyl-CoA pools and shifts endothelial cells toward a mesenchymal, pro-fibrotic state. Given that RCC frequently arises in kidneys with chronic injury, inflammation, or metabolic dysfunction, FAO-regulated endothelial plasticity may represent an underappreciated link connecting lipid metabolism, sex differences, and renal cancer biology. Additionally, exposome-level environmental factors may modulate these metabolic differences [12]. Endocrine-disrupting chemicals, such as phthalates and bisphenols, are pervasive in modern environments and can exert measurable metabolic effects even at low doses. Importantly, many endocrine disruptors interact with androgen and estrogen receptor signaling [12], raising the possibility that sex-specific hormonal pathways may magnify the impact of environmental exposures.

Within this context, acetate has emerged as a promising meta­bolic modulator capable of mitigating kidney injury [9]. In the present study, acetate supplementation primarily restored acetyl-CoA levels and suppressed EndoMT-associated gene expression; however, whether acetate treatment improves renal fibrosis or functional outcomes *in vivo* remains to be determined. Future studies directly evaluating the impact of acetyl-CoA restoration on kidney injury and fibrosis will be essential to establish the therapeutic relevance of this pathway. As an alternative carbon source during metabolic stress, acetate suppresses pro-fibrotic signaling and preserves endothelial stability [6]. These properties position acetate at the intersection of metabolism and endothelial biology. This suggests that modulating acetate pathways may offer a novel therapeutic avenue for preventing or reversing renal injury and fibrosis. Targeted proteomic analyses comparing FAO-deficient and acetate-rescued endothelial states may identify FAO-responsive endothelial protein markers involved in metabolic stress, EndoMT progression, and early renal injury. The restoration of such markers may parallel therapeutic normalization of endothelial metabolic health and concomitant reduction in disease risk. Although additional studies are needed to define optimal delivery strategies and potential sex-specific effects, the accumulating data highlight the translational potential of acetate in kidney disease.

In conclusion, our study identifies endothelial FAO as a critical metabolic checkpoint that restrains EndoMT, preserves endothelial identity, and protects against renal injury and fibrosis. The observed sex-specific differences in functional decline further suggest that endothelial metabolic programs may contribute to the well-known disparity in renal disease and RCC severity between males and females. More broadly, these findings implicate FAO-derived acetyl-CoA as a central regulator of endothelial fate and highlight lipid metabolism as a potential therapeutic target for mitigating chronic kidney injury.

## Limitations of the study

A limitation of this study is the use of VE-cadherin–Cre, which induces endothelial *Cpt2* deletion throughout the vasculature and precludes definitive separation of renal endothelial cell–autonomous effects from secondary systemic consequences. In addition, ultrastructural characterization by electron microscopy was not performed and represents an important focus for future studies. Other limitations include the use of whole-organ readouts that cannot yet distinguish contributions from specific vascular beds, the lack of direct mechanistic dissection of sex hormone or sex chromosome effects on endothelial FAO, and the absence of longitudinal functional studies in aged cohorts, where fibrosis and proteinuria may become more pronounced. Future studies integrating single-cell multi-omics, endothelial-specific metabolic flux analyses, and sex-stratified approaches will be essential to define how FAO intersects with sex biology to regulate kidney pathology and to assess whether these mechanisms have any relevance to RCC-associated microenvironments.

## Supplementary Material

loag017_Supplementary_Data

## Data Availability

All data generated or analyzed during this study are included in this article and its supplementary information files.

## References

[1] Houten SM , Violante S, Ventura FV et al Annu Rev Physiol 2016;78:23–44. 26474213 10.1146/annurev-physiol-021115-105045

[2] Kang HM , Ahn SH, Choi P et al Nat Med 2015;21:37–46. 25419705 10.1038/nm.3762 PMC4444078

[3] Xiong J. Trends Endocrinol Metab 2018;29:809–13. 30314937 10.1016/j.tem.2018.09.006 PMC12866293

[4] Xiong J. Trends Biochem Sci 2018;43:854–7. 29735398 10.1016/j.tibs.2018.04.006 PMC6204300

[5] Nyman LR , Cox KB, Hoppel CL et al Mol Genet Metab 2005;86:179–87. 16169268 10.1016/j.ymgme.2005.07.021

[6] Xiong J , Kawagishi H, Yan Y et al Mol Cell 2018;69:689–98.e7. 29429925 10.1016/j.molcel.2018.01.010 PMC5816688

[7] Xiong J. Protein Cell 2015;6:547–50. 26099567 10.1007/s13238-015-0183-z PMC4506284

[8] Bronson R , Lyu J, Xiong J. G3 (Bethesda) 2023;13:jkad243. 37857450 10.1093/g3journal/jkad243 PMC10700110

[9] Xiong J. Serican J Med 2024;1:23080. 42111959 10.17161/sjm.v1i1.23080 PMC13155442

[10] Lucca I , Klatte T, Fajkovic H et al Nat Rev Urol 2015;12:585–92. 26436686 10.1038/nrurol.2015.232

[11] Jacobs ME , de Vries DK, Engelse MA et al Nephrol Dial Transplant 2024;39:752–60. 37968135 10.1093/ndt/gfad238

[12] Kahn LG , Philippat C, Nakayama SF et al Lancet Diabetes Endocrinol 2020;8:703–18. 32707118 10.1016/S2213-8587(20)30129-7 PMC7437820

