# Supplementary material for: Endothelial CPT2 loss impairs fatty acid oxidation and promotes renal fibrosis
Source: Life Metab. 2026 Jun 13;5(4):loag017. doi: 10.1093/lifemeta/loag017 (PMC13342721; doi:10.1093/lifemeta/loag017)
Supplement: loag017_Supplementary_Data [file loag017_supplementary_data.docx]

Supplementary Materials for

**Endothelial CPT2 loss impairs fatty acid oxidation and promotes renal fibrosis**

**Materials and methods**

**Animals**

*Cpt2*flox/flox mice [13] were crossed with VE-cadherin–Cre transgenic mice (Jackson Laboratory) [14] to generate endothelial-specific *Cpt2* knockout mice (*Cpt2*E-KO) [6]. To enable lineage tracing, *Cpt2*E-KO mice were further crossed with R26-mT/mG Cre reporter mice (Jackson Laboratory) [15], yielding *Cpt2*E-KO; mT/mG mice. All mice were on a C57BL/6J background and genotyped using standard PCR. Unless otherwise indicated, experiments included both male and female mice, and each cohort represented an independent set of animals.

**Cells**

Mouse endothelial cells were isolated and cultured as previously described [16–18]. Briefly, three mouse lungs were dissected, minced, and digested in PBS containing 0.1% type I collagenase (ThermoFisher Scientific) at 37 °C for 1 h. The suspension was filtered, collected by centrifugation, and plated on T-75 flasks coated with fibronectin (Sigma-Aldrich, F4759), gelatin (G1393), and collagen (C4243). Sequential magnetic sorting was performed to remove macrophages using sheep anti-rat IgG Dynabeads coated with rat anti-mouse CD16/CD32 (BD Biosciences, 553142), followed by positive selection of endothelial cells using anti-CD102 antibody (BD Biosciences, 553326). Purity was confirmed by APC-conjugated anti-mouse CD31 (PECAM1) staining (BD Biosciences, 551262, 1:70) and analyzed with a flow cytometer. Freshly isolated endothelial cells were maintained in DMEM/F12 (ThermoFisher Scientific) supplemented with 20% FBS, 2 mmol/L L-glutamine, 0.1 mg/mL heparin, 0.05 mg/mL endothelial growth supplement, and 100 U/mL penicillin/streptomycin. Cells were used between passages 1 and 4. Human glomerular microvascular endothelial cells (HGMECs; iXCells Biotechnologies, Cat. No. 10HU-061) were cultured in Complete Endothelial Cell Medium (Cell Biologics, Cat. No. H1168) supplemented with the Endothelial Cell Medium Supplement Kit, containing VEGF (0.5 mL), heparin (0.5 mL), EGF (0.5 mL), FGF (0.5 mL), hydrocortisone (0.5 mL), antibiotic–antimycotic solution (5.0 mL), and fetal bovine serum (25.0 mL). The commercially purchased human endothelial cells were used between passages 3 and 8.

**Chemicals, plasmids, and lentiviral vectors**

Lentiviral vectors for mouse *Cpt2* overexpression (EX-Mm34188-Lv105) and the corresponding vector control (EX-NEG-Lv105) were purchased from GeneCopoeia, while lentiviral constructs for stable shRNA-mediated

knockdown of human gene expression included *CPT2*-targeting shRNA (sh*CPT2*) and a scrambled control shRNA (shSCR; Addgene, plasmid #1864). Plasmids were purified using the ZymoPURE Plasmid Miniprep Kit (Zymo Research, D4212), and lentiviral particles were produced in HEK293T cells (Takara Bio) by co-transfecting lentiviral vectors with the packaging plasmids psPAX2 and pMD2.G using polyethylenimine (PEI). Viral supernatants were collected 48 h after transfection, filtered through 0.45-μm filters, and concentrated using the Lenti-X Concentrator (Takara Bio). Endothelial cells were infected with lentiviral particles in the presence of 8 μg/mL Polybrene (Santa Cruz Biotechnology) overnight, after which the medium was replaced with fresh culture medium and cells were used for subsequent experiments. Sodium acetate (Sigma-Aldrich) was added where indicated at 40 mmol/L, a concentration similar to what has been previously employed consistent with previous studies [6, 19–22].

**Western blot analysis**

Cells were lysed in RIPA buffer (50 mmol/L Tris-HCl, pH 7.4, 150 mmol/L NaCl, 1% NP-40, 0.5% sodium deoxycholate, and 0.1% SDS; Boston BioProducts, BP-115) or MCE WB/IP Lysis Buffer (MedChemExpress, HY- K1000) supplemented with protease inhibitors (Roche, 11836170001). Lysates were mixed with 4× Laemmli buffer (Bio-Rad, 1610747) and boiled at 95 °C for 10 min. Proteins were separated by SDS-PAGE and transferred to nitrocellulose membranes using the Trans-Blot Turbo system (Bio-Rad). Membranes were blocked for 1 h at room temperature and incubated with primary antibodies: anti-CPT2 (Abcam, ab181114), anti-GAPDH (ABclonal, A19056; Proteintech, 60004-1-IG), anti-KIM-1 (Novus Biologicals, NBP1-76701), and anti-HSP60 (GeneTex, GTX110089; Santa Cruz Biotechnology, sc-1052). After 5 min washes with TBST (TBS + 0.1% Tween-20) three times, membranes were incubated with near-infrared IR Dye secondary antibodies for 1 h at room temperature. Protein detection and quantification were performed using Odyssey CLx or DLx Imagers and Image Studio Lite v6.0 (LI-COR). Expression levels were normalized to loading controls: GAPDH for whole-cell lysates and HSP60 for mitochondrial extracts.

**Real-time quantitative PCR (RT-qPCR)**

Total RNA from cultured mouse endothelial cells and homogenized mouse kidney tissues (2–7-month-old mice) was extracted using the Direct-zol RNA Miniprep Plus Kit (R2073; Zymo Research). DNase I treatment was performed during purification to remove genomic DNA. Complementary DNA was prepared using iScript cDNA Synthesis Kit (BioRad). RT-qPCR was performed on an QuantStudio 5 (ThermoFisher Scientific) or MxP3005P (Stratagene) real-time PCR system using FastStart Universal SYBR Green Master Mix (Roche) or SYBR Green qPCR Master Mix (GlpBio, GK10002) according to the manufacturer’s instructions. The following primers were used to measure gene expression: 18S rRNA, forward primer, 5’-GTAACCCGTTGAACCCCATT-3’, and reverse primer, 5’-CCATCCAATCGGTAGTAGCG-3’; mouse *Cd44*, forward primer, 5’-TCGATTTGAATGTAACCTGCCG-3’, and reverse primer, 5’- CAGTCCGGGAGATACTGTAGC-3’; mouse *Cdh2*, forward primer, 5’-AGCGCAGTCTTACCGAAGG-3’, and reverse primer, 5’-TCGCTGCTTTCATACTGAACTTT-3’; mouse *Cpt2*, forward primer, 5’- ATGCACTACCAGGACAGCCT-3’, and reverse primer, 5’-TGGCTGTCATTCAAGAGAGG-3’; mouse *Gapdh*, forward primer, 5’-CGTCCCGTAGACAAAATGGT-3’, and reverse primer, 5’- TTGATGGCAACAATCTCCAC-3’; mouse *KIM-1*, forward primer, 5’- ACATATCGTGGAATCACAACGAC-3’, and reverse primer, 5’-ACAAGCAGAAGATGGGCATTG-3’; mouse *Zeb2*, forward primer, 5’-ATTGCACATCAGACTTTGAGGAA-3’, and reverse primer, 5’- ATAATGGCCGTGTCGCTTCG-3’; human *ANGPT2*, forward primer, 5’- ATTCAGCGACGTGAGGATGGCA-3’, and reverse primer, 5’-GCACATAGCGTTGCTGATTAGTC-3’; human *CCL2*, forward primer, 5’-ATCACCAGCAGCAAGTGTC-3’, and reverse primer, 5’- AGGTGGTCCATGGAATCCTG-3’; human *CD31*, forward primer, 5’-AAGTGGAGTCCAGCCGCATATC-3’, and reverse primer, 5’-ATGGAGCAGGACAGGTTCAGTC-3’; human *CPT2*, forward primer, 5’- TGCCGTCCACTTTGAGCACT-3’, and reverse primer, 5’-GGGGTCTGAGTGCTGTCTTT-3’; human *GAPDH*, forward primer, 5’-AAGGTGAAGGTCGGAGTCAA-3’, and reverse primer, 5’- AATGAAGGGGTCATTGATGG-3’; human *ICAM1*, forward primer, 5’- AGCGGCTGACGTGTGCAGTAAT-3’, and reverse primer, 5’-TCTGAGACCTCTGGCTTCGTCA-3’; human *IL6*, forward primer, 5’-AGACAGCCACTCACCTCTTCAG-3’, and reverse primer, 5’- TTCTGCCAGTGCCTCTTTGCTG-3’; human *VCAM1*, forward primer, 5’- GATTCTGTGCCCACAGTAAGGC-3’, and reverse primer, 5’-TGGTCACAGAGCCACCTTCTTG-3’.

**Cellular imaging and quantitative image analysis**

For mitochondrial staining, cells were seeded onto 30 mm × 10 mm glass-bottom tissue culture dishes (CELLTREAT Scientific Products, Cat. No. 229632) allowed to attach overnight, and processed as described below. Following the indicated treatments, live cells were incubated with MitoTracker Green (Thermo Fisher Scientific, M7514) at 37 °C for 30 min to label mitochondria and Hoechst 33342 (Thermo Fisher Scientific, H1399) to stain nuclei, according to the manufacturer’s instructions. Cells were imaged using a laser-scanning confocal microscope (STELLARIS 8; Leica Microsystems) equipped with a 40× oil-immersion objective, with identical acquisition settings applied across conditions. Mitochondrial morphology was quantified using the Mitochondrial Network Analysis (MiNA) toolset in ImageJ [23]. Raw images were converted to 8-bit grayscale, the background was subtracted, and noise was reduced by median filtering. Images were then thresholded using the Otsu algorithm to generate binary mitochondrial masks, skeletonized, and analyzed using the Analyze Skeleton plugin. Quantitative parameters included average branch length and total branch number, and 17 cells from independent experiments were analyzed per condition.

For CD31 immunofluorescence, cells were seeded and treated as described above, washed with PBS, and fixed with 4% paraformaldehyde for 15 min at room temperature. Cells were permeabilized with 0.1% Triton X-100 for 10 min, blocked with 5% bovine serum albumin (BSA) in PBS for 1 h at room temperature, and incubated overnight at 4 °C with an anti-CD31 primary antibody (GeneTex, Cat. No. GTX642317). Cells were then incubated with Alexa Fluor 488-conjugated secondary antibody (Thermo Fisher Scientific, A-11008) for 1 h at room temperature, followed by nuclear counterstaining with DAPI (4′,6-diamidino-2-phenylindole). Confocal images were acquired using the same microscope and objective. Fluorescence intensity was quantified using ImageJ by manually selecting regions of interest for individual cells, subtracting background fluorescence, and calculating mean fluorescence intensity. Ten cells from seven randomly selected fields were analyzed per condition.

**Metabolic assays**

Fatty acid oxidation (FAO) in *Cpt2*E-WT and *Cpt2*E-KO endothelial cells was assessed using the XF96 extracellular flux analyzer (Seahorse Bioscience). Endothelial cells (2 × 104 per well) were seeded in XF96 microplates coated with fibronectin, gelatin, and collagen. Utility plate wells were filled with 200 µL XF Calibrant Solution and incubated overnight at 37 °C in a humidified, non-CO₂ incubator to hydrate the sensors. On the day of the assay, the medium was replaced with FAO assay medium (111 mmol/L NaCl, 4.7 mmol/L KCl, 1.25 mmol/L CaCl₂, 2.0 mmol/L MgSO₄, 1.2 mmol/L Na₂HPO₄, 2.5 mmol/L glucose, 0.5 mmol/L carnitine, and 5 mmol/L HEPES) for 45 min. Cells were then treated with either palmitate-BSA (166.7 µmol/L palmitate conjugated to 28.3 mmol/L BSA) or BSA alone (28.3 mmol/L). Oxygen consumption rate (OCR) was measured using the XF Cell Mito Stress Test and Wave software. Acetyl-CoA levels were quantified using the PicoProbe Acetyl-CoA Fluorometric Assay Kit (BioVision). ATP levels were measured with the Luminescent ATP Detection Assay Kit (Abcam, ab113849). For ATP detection, 4,000 cells per well were plated in 96-well black, flat-bottom microplates (Greiner Bio-One) two days before the assay. Cells were lysed, and ATP was stabilized with 50 µL of the kit-provided detergent and shaken for 5 min. After adding 50 µL of substrate solution, plates were shaken for another 5 min, incubated in the dark for 10 min, and luminescence was recorded using a luminescence counter.

**Fluorescence-activated cell sorting (FACS) analysis of renal EndoMT**

Kidney cells were isolated from 2–13-month-old, sex- and age-matched *Cpt2*E-WT; mT/mG or *Cpt2*E-KO; mT/mG [24]. Kidneys were dissected, minced, and digested in PBS with 0.09% type II collagenase and 0.25 U/mL dispase at 37 °C for 1 h. DNase I (7.5 µg/mL, Sigma) was included to reduce DNA viscosity. Cells were purified by Ficoll-Paque PLUS centrifugation (400 g, 20 min, 18 °C, no brake) to remove red blood cells, granulocytes, and debris. Cells were stained with APC–CD31 (BD Biosciences, 551262) and BV785–CD45 (BioLegend, 103149) antibodies for 30 min at 4 °C. After PBS washes, cells were fixed in 2% paraformaldehyde (10 min), permeabilized with 0.1% Triton X-100 (10 min), and stained with APC-Cy7–α- SMA antibody (Abcore, AC12-0159-05; 30 min, 4 °C). EndoMT was defined as GFP+, tdTomato–, CD45–, CD31+, α-SMA+ cells. Data were acquired and analyzed using a flow cytometer.

**Analyses of renal function and histology**

Urinary albumin-to-creatinine ratios were measured in 10–14-month-old male mice using morning spot urine samples. Albumin and creatinine levels were quantified with Albuwell M and Creatinine Companion ELISA Strip Plates (Exocell). For fibrosis analysis, 5-µm sections of paraffin-embedded kidneys from 13–16-month-old male mice were stained with Masson’s trichrome. Slides were imaged using a NanoZoomer 2.0-RS digital scanner, and fibrotic areas were quantified with MetaMorph software. Imaging and analysis settings were kept constant across groups, and all assessments were performed in a blinded manner.

**Statistical analysis**

Unless otherwise specified, differences between control and experimental groups were evaluated using unpaired, two-tailed Student’s *t*-tests in GraphPad Prism 10 (v10.2.3; GraphPad Software). Data are presented as mean ± standard error of the mean (SEM), and *P*-values < 0.05 were considered statistically significant.

**References**

1. Lee J, Ellis JM, Wolfgang MJ. Adipose fatty acid oxidation is required for thermogenesis and potentiates oxidative stress-induced inflammation. *Cell Rep* 2015;10(2):266-79.
2. Alva JA, Zovein AC, Monvoisin A *et al*. VE-Cadherin-Cre-recombinase transgenic mouse: a tool for lineage analysis and gene deletion in endothelial cells. *Dev Dyn* 2006;235(3):759-67.
3. Muzumdar MD, Tasic B, Miyamichi K *et al*. A global double-fluorescent Cre reporter mouse. *Genesis* 2007;45(9):593-605.
4. Reynolds LE, Hodivala-Dilke KM. Primary mouse endothelial cell culture for assays of angiogenesis. *Methods Mol Med* 2006;120:503-9.
5. Xiong J, Hou J. Apical Resection Mouse Model to Study Early Mammalian Heart Regeneration. *J Vis Exp* 2016(107):e53488.
6. Xiong J. BMPR2 spruces up the endothelium in pulmonary hypertension. *Protein Cell* 2015;6(10):703-8.
7. Schoors S, Bruning U, Missiaen R *et al*. Fatty acid carbon is essential for dNTP synthesis in endothelial cells. *Nature* 2015;520(7546):192-7.
8. Lyu J, Li Z, Roberts JP *et al*. The short-chain fatty acid acetate coordinates with CD30 to modulate T-cell survival. *Mol Biol Cell* 2023;34(8):br11.
9. Lyu J, Pirooznia M, Li Y *et al*. The short-chain fatty acid acetate modulates epithelial-to-mesenchymal transition. *Mol Biol Cell* 2022;33(8):br13.
10. Jing X, Lyu J, Xiong J. Acetate regulates GAPDH acetylation and T helper 1 cell differentiation. *Mol Biol Cell* 2023;34(7):br10.
11. Valente AJ, Maddalena LA, Robb EL *et al*. A simple ImageJ macro tool for analyzing mitochondrial network morphology in mammalian cell culture. *Acta Histochem* 2017;119(3):315-26.
12. van Beijnum JR, Rousch M, Castermans K *et al*. Isolation of endothelial cells from fresh tissues. *Nat Protoc* 2008;3(6):1085-91.

**Supplementary Figures**





**Supplementary Figure S1** Metabolic and functional consequences of endothelial CPT2 loss. (a) Basal oxygen consumption rate (OCR) in endothelial cells treated with BSA or palmitate-BSA (Palm-BSA) (n = 3 technical replicates per condition; representative of two independent experiments). (b) Relative acetyl-CoA levels in endothelial cells with or without acetate supplementation (n = 3 independent experiments). (c) Strategy for quantifying EndoMT as CD31+/α-SMA+ events within GFP+, CD45–, tdTomato–renal endothelial cells isolated from Cpt2E-KO; mT/mG mice. Approximately 6% of renal cells were GFP+. (d) FACS analysis of CD31+/α-SMA+ double-positive endothelial cells in Cpt2E-KO; mT/mG kidneys. (e) Quantification showing an approximately 30% increase in CD31+/α-SMA+ cells in Cpt2E-KO kidneys compared with controls (fold change 1.30 ± 0.12; n = 4 pairs). (f) Urine albumin-to-creatinine ratios in 10–14-month-old Cpt2E-WT and Cpt2E-KO mice (n = 16 total; 8 males and 8 females per genotype). (g and h) Representative confocal immunofluorescence images (g) showing CD31 (green) and nuclei (blue) in control (Ctrl) and CPT2 knockdown (shCPT2) human glomerular microvascular endothelial cells (HGMECs). CPT2 knockdown markedly reduced CD31 expression and disrupted endothelial junctional organization. Scale bar, 20 μm. Quantification (h) of CD31 mean fluorescence intensity. (i) qPCR analysis in HGMECs showing that CPT2 knockdown (shCPT2) significantly reduces endogenous CPT2 mRNA expression and markedly increases inflammatory gene expression, including IL6, CCL2, VCAM1, and ICAM1. Acetate supplementation partially attenuates the induction of these inflammatory markers. Data represent mean ± SEM. Statistical significance was assessed by one-way ANOVA with Tukey’s multiple comparisons test (a, b, and i). *P < 0.05; **P < 0.01; ***P < 0.001; ns, not significant.
